# Features of Neural Network Formation and Their Functions in Primary Hippocampal Cultures in the Context of Chronic TrkB Receptor System Influence

**DOI:** 10.3389/fphys.2018.01925

**Published:** 2019-01-10

**Authors:** Tatiana A. Mishchenko, Elena V. Mitroshina, Alexandra V. Usenko, Natalia V. Voronova, Tatiana A. Astrakhanova, Olesya M. Shirokova, Innokentiy A. Kastalskiy, Maria V. Vedunova

**Affiliations:** ^1^Department of Neurotechnology, Institute of Biology and Biomedicine, National Research Lobachevsky State University of Nizhni Novgorod, Nizhny Novgorod, Russia; ^2^Molecular and Cell Technologies Group, Central Scientific Research Laboratory, Privolzhsky Research Medical University, Nizhny Novgorod, Russia

**Keywords:** TrkB receptor, brain-derived neurotrophic factor (BDNF), primary hippocampal cell cultures, neural networks, multielectrode arrays, calcium imaging, mitochondrial functional activity

## Abstract

Discovering the mechanisms underlying homeostatic regulation in brain neural network formation and stability processes is one of the most urgent tasks in modern neuroscience. Brain-derived neurotrophic factor (BDNF) and the tropomyosin-related kinase B (TrkB) receptor system have long been considered the main regulators of neuronal survival and differentiation. The elucidation of methods for studying neural network activity makes investigating the complex mechanisms underlying neural network structure reorganization during development and detecting new mechanisms for neuronal activity remodeling possible. In this *in vitro* study, we investigated the effects of chronic BDNF (the main TrkB stimulator) and ANA-12 (a TrkB receptor system blocker) administration on the formation of neural-glial networks. The formation of spontaneous bioelectrical activity and functional neural structure depend on TrkB receptors, and blocking TrkB receptors inhibits full bioelectrical activity development. Cross-correlation analysis demonstrated the decisive role of TrkB in the formation and “strengths” of activity centers. Even though an appropriate ANA-12 concentration is non-toxic to nerve cells, numerous cells in culture medium containing this reagent do not exhibit metabolic activity and are not functionally involved in signal transmission processes. Electron microscopy studies revealed that chronically influencing the TrkB receptor system significantly alters synaptic and mitochondrial apparatus capture in cells, and functional analysis of mitochondrial activity confirmed these findings. Because knowledge of interactions between TrkB-mediated regulation and the mitochondrial state under normal conditions is rather limited, data on these relationships are particularly interesting and require further investigation. Thus, we assume that the molecular cascades mediated by TrkB actively participate in the formation of functionally complete brain neural networks.

## Introduction

Neural networks are currently considered the minimal functional unit of the central nervous system (CNS), and formation of the brain neural networks responsible for processing and transmitting information is a complex process characterized by a number of critical stages (Shirokova et al., 2013; Yuste, 2015). Each stage has its own peculiarities, and issues in any of these stages lead to the development of a functionally inferior structure. Even though the brain structure is formed before birth, its full development is mainly determined by the nature of postpartum stimuli from the environment. External stimuli modulate functional brain maturation and neurogenesis in adulthood (Lledo et al., 2006; de Almeida et al., 2013; Sailor et al., 2017). Notably, the formation of synaptic contacts between neurons continues throughout life. Organization of a stable neural network structure in the brain and reconsolidation of synaptic contacts under stress conditions are associated with the activation of numerous intracellular signaling cascades (de Almeida et al., 2013; Yu and Yu, 2017). Searching for endogenous compounds involved in the formation of complex spatial and functional neural networks is one of the most urgent tasks in modern neuroscience.

Brain-derived neurotrophic factor (BDNF) is a promising signaling molecule that may have a generalized effect on the formation and reconsolidation of neural networks. BDNF is involved in the regulation of neurogenesis, neuronal development and survival (Martin and Finsterwald, 2011; Park and Poo, 2013; Skaper, 2018); it also plays a crucial role in early neuronal differentiation, synaptic development, neural outgrowth, mature neuron survival, and synaptic plasticity (Douglas-Escobar et al., 2012; Leal et al., 2015; Kowiański et al., 2018; von Bohlen Und Halbach and von Bohlen Und Halbach, 2018).

The main functions of BDNF are mediated by its interaction with the tropomyosin-related kinase B (TrkB) receptor (Patapoutian and Reichardt, 2001; Skaper, 2018) and the possibility of intracellular signaling cascade activation, which can indirectly affect synaptic transmission and synaptic contact formation, thus determining the neural network structure (Ohira and Hayashi, 2009; Guo et al., 2014; Mitre et al., 2017). TrkB receptors are normally localized within vesicles inside the cell and translocate to the plasma membrane through neuronal activity (Meyer-Franke et al., 1998; Du et al., 2000). These data are consistent with a central role of neurotrophins as mediators of activity-dependent plasticity (Poo, 2001; Lu et al., 2014). BDNF plays a key role in mediating activity-induced long-term potentiation (LTP) (Leal et al., 2014). The early effects of BDNF result from the modification (e.g., protein phosphorylation) of components already present at the synapse, while the long-term effects arise from the modification of translational activity at the synapse and changes in transcription. High-frequency stimulation that induces LTP increases BDNF production (Castrén et al., 1993). Furthermore, BDNF increases neurotransmitter release and promotes synaptic transmission and LTP (Panja and Bramham, 2014; Kuipers et al., 2016). Thus, it is reasonable to assume that the effects exerted by BDNF on synaptic plasticity are TrkB mediated.

Investigating neural network formation at different levels of neuron-glial system organization under chronic influence of the TrkB receptor system during development is of particular interest. Application of mathematical methods for biological data analysis may help reveal the features of neural network internal structure formation and the influence of TrkB signaling on the nerve impulse transmission process as well as predict the effects of endogenous BDNF dynamics on neural networks.

Our present study is devoted to investigating the features of neural network formation and functions in primary hippocampal cultures in the context of chronic BDNF application and TrkB receptor blockage.

## Materials and Methods

### Ethics Statement

All experimental protocols utilized in this study were approved by the Bioethics Committee of Lobachevsky University and carried out in accordance to Act708n (23 08 2010) of the Russian Federation National Ministry of Public Health, which states the rules of laboratory practice for the care and use of laboratory animals, and the Council Directive 2010/63 EU of the European Parliament (September 22, 2010) on the protection of animals used for scientific purposes. C57BL/6J mice were killed by cervical vertebra dislocation, and their embryos were then surgically removed and sacrificed by decapitation.

### Experimental Scheme

**FIGURE 1:**
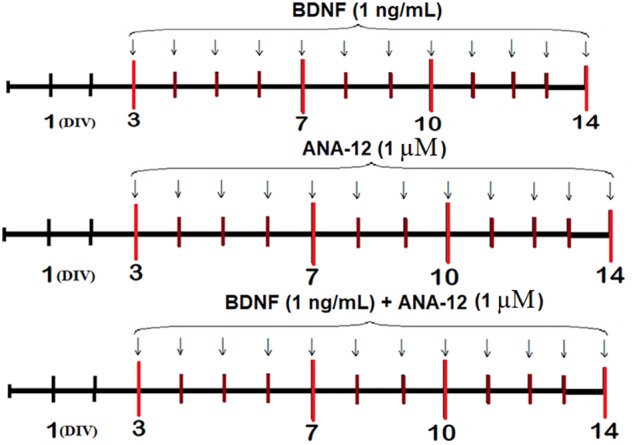
Scheme of the experiment.

Recombinant BDNF (1 ng/mL, Merck, GF301, Germany), ANA-12 (a selective TrkB receptor blocker, 1 μM, Sigma-Aldrich, SML0209, Germany) or BDNF (1 ng/mL) and ANA-12 (1 μM) in combination were added to the culture medium daily beginning on the third day of culture development *in vitro* (DIV) (Figure 1).

Analyses of spontaneous bioelectrical and calcium activities, which included defining the internal neural network structure, morphological studies, and evaluation of the dynamics of functional characteristics of the mitochondrial respiratory chain in neural cells, were performed on DIV 7, 10, and 14.

### Cell Culture

Hippocampal cells were obtained from mouse embryos (day 18 of gestation) and cultivated on multielectrode arrays (MEAs) (Multichannel Systems, Germany) or coverslips pretreated with polyethyleneimine solution (1 mg/mL) (Sigma-Aldrich, P3143) according to a previously developed protocol (Vedunova et al., 2013). Hippocampi were dissected in Ca^2+^- and Mg^2+^-free phosphate buffered saline (PBS-minus) followed by 20 min of enzymatic treatment with 0.25% trypsin-ethylenediaminetetraacetic acid (EDTA, Invitrogen, 25200-056, United States). Next, the cells were carefully suspended and centrifuged at 1,000 rotations per min (rpm) for 3 min. The cell pellet was immediately resuspended in neurobasal medium (Invitrogen, 21103-049) supplemented with 2% B27 (Invitrogen, 17504-044), 0.5 mM L-glutamine (Invitrogen, 25030-024), and 5% fetal bovine serum (PanEco, K055, Russia). Dissociated cells were plated on MEAs and coverslips at an approximate initial density of 9,000 cells/mm^2^. After 24 h, 50% of the culture medium was replaced with neurobasal medium containing a lower concentration of fetal bovine serum (0.4%). Fifty percent of the medium was changed every third day, and cell viability was maintained under constant conditions of 35.5°C, 5% CO_2_ and a humidified atmosphere in a cell culture incubator.

### Cell Viability Detection

To determine the viability of dissociated hippocampal cells upon application of different concentrations of ANA-12, we estimated the ratio of the number of dead cells stained with propidium iodide (Sigma-Aldrich, P4170) to the total number of cells stained with bisbenzimide (Invitrogen, H3570). Propidium iodide and bisbenzimide at concentrations of 5 μg/mL and 1 μg/mL, respectively, were added to the culture medium 30 min before the viability was measured (Vedunova et al., 2015). Cells were observed under a Leica DMIL HC inverted fluorescence microscope (Leica, Germany) with a 10×/0.2Ph1 objective.

### Electrophysiological Methods

Extracellular potentials were collected using 59 planar TiN electrodes integrated into the USB-MEA-120 system (Multichannel system, Germany). The MEAs had 59 electrodes (8 × 8 grid) with a diameter of 30 μm and were spaced 200 μm apart. Data were recorded simultaneously from 59 channels at a sampling rate of 20 kHz/channel. All signaling and statistical analyses were performed using custom-made software (MATLAB^®^6.0, Natick, MA, United States).

Small network bursts were detected by calculating the total spiking rate (TSR), which considered the total number of spikes from all electrodes within 50-ms time bins. The criterion for a small network burst was the rapid appearance of a large number of spikes over four electrodes within a small (50 ms) time bin (Pimashkin et al., 2011; Vedunova et al., 2013; Gladkov et al., 2018).

#### Spike Detection

The recorded extracellular action potentials were detected by threshold calculations using the signal median as follows:

$$T=N_{s}\sigma ,\sigma =median\left(\frac{\left|x\right|}{0.6745}\right)$$

where *x* is the bandpass-filtered (0.3–8 kHz) data signal, σ is an estimate of the standard deviation of the signal without spikes, and N_s_ is the spike detection coefficient that determines the detection threshold. Threshold estimations based on the median of the signal in the form of Eq. (1) are less dependent on the frequency of the spikes than threshold estimates based on the standard deviation during signal processing. The coefficient 0.6745 in Eq. (1) was used to normalize the median of the absolute signal to the standard deviation. N_s_ = 4 was used for all data, which allowed the reliable detection of spikes with amplitudes greater than 20 μV. The minimal interspike interval was set to 1 ms. Detected spikes were plotted using raster diagrams (Quiroga et al., 2004; Pimashkin et al., 2011).

#### Small Burst Detection

We recorded spontaneous burst activity to analyze the effects of chronic BDNF and ANA-12 application on the functional characteristics of neural networks in primary hippocampal cultures. The TSR was determined by counting the total number of spikes from all electrodes within 50-ms time bins for small network burst detection. The rapid emergence of a large number of spikes over multiple electrodes within a small (50 ms) time bin was used as the criterion for a small network burst. Spontaneous activity in the cultures corresponded to the basal stochastic activity, which was observed in fractions of cells together with short burst episodes. The spike trains (approximately 1 spike per 10–100 ms) were considered to represent basal activity. To reveal bursts, we used a threshold detection based on the statistical characteristics of the spontaneous activity TSR(t). The burst threshold was set to TBurst = 0.1 × σ TSR, where σ TSR was the standard deviation of TSR(t). The burst detection threshold coefficient was empirically set to 0.1, yielding the best estimate for the burst initiation and end points according to the raster diagram. Simulations of bursts with frequencies up to 5 Hz revealed that the estimated burst durations were within 10% of the actual values. Statistical analysis of the bursting activity characteristics was performed using analysis of variance (ANOVA, *p* < 0.05) (Pimashkin et al., 2011; Vedunova et al., 2013).

#### Cross-Correlation Method and Graphs

The dataset, obtained from spontaneous bioelectrical network activity recordings, was represented as a raster plot. The existence of functional connections between neuronal groups was not obvious based on visual analysis. The network graph method was then used to detect the neuronal groups.

First, to assess the degree of synchronization between all pairs of cells, considering axonal delays, we calculated the proportion of transmitted spikes. This measure is an analog of the cross-correlation coefficient of continuous signals. According to this method, the number of “delayed synchronous” spikes was calculated. These spikes had to be recorded from both channels within a tolerance interval of δ for which the time delay τ between the centers of the spikes was proportional to the distance between the electrodes. The number of delayed synchronous spikes was normalized by the number of spikes received by the postsynaptic neuron *n_j_*. Thus, the cross-correlation matrix was calculated using the following formula:

$$C_{ij}=\frac{n_{sunchr,ij}}{n_{j}}$$

we then selected the largest 5% of *C_ij_* coefficients and defined a set of indices, i.e., hubs of cells with a maximum number of functionally active connections. In addition, for each hub “*i*,” we calculated the number of connections to index *i* within the array *C_ij_*.

Next, the graph was constructed. The vertex size was proportional to the number of significant connections, and the edge of the graph corresponded to the functional connections of spikes transferred from one neuron to another at individual time points for each pair of axonal delays, i.e., *τ* ± δ/2 (Shishkina et al., 2018).

The cross-correlation method and graphs allow the detection of hubs, i.e., elements with the maximum number of functionally active connections, and show the dynamic changes occurring in the network in short- and long-term periods. The hub coefficient was calculated as the ratio of the number of connections of an electrode to its total number in the graph; therefore, it characterized the importance of a group of neurons located at one electrode to network activity. The hub coefficient allows an estimation of changes in the significance of each electrode in an MEA.

### Ca^2+^ Imaging

To conduct functional calcium imaging, we dissolved Oregon Green 488 BAPTA-1 AM (OGB-1) (0.4 μM, Invitrogen, O-6807) in dimethylsulfoxide (DMSO) (Sigma-Aldrich, D8418) with 4% pluronic F-127 (Invitrogen, P-3000 MP) and then added it to the culture medium for 40 min. After incubation to allow full absorption of OGB-1 molecules by the cells, the cells were washed with dye-free medium for 15 min. A confocal laser-scanning microscope (Zeiss LSM 510, Germany) with a W Plan-Apochromat 20×/1.0 objective was used to visualize spontaneous calcium activity in the dissociated cultures.

Cytosolic Ca^2+^ was visualized via OGB-1 excitation with an Argon laser at 488 nm and emission detection with a 500–530 nm filter. Time series of 256 × 256 pixel images capturing 420 μm × 420 μm fields of view were recorded at 4 Hz. A confocal pinhole of 1 airy unit was used to obtain an axial optical slice resolution of 1.6 μm.

Quantitative evaluation of Ca^2+^ transients was performed off-line using custom-made software in C++ Builder. Cell regions from fluorescent images were manually selected. The Ca^2+^ fluorescence of each cell in each frame was calculated as the average fluorescence intensity (F, relative units from 0 to 255) of the pixels within the defined cell region. Single Ca^2+^ signals were identified using the following algorithm. First, each trace from all of the cells was filtered by averaging two neighboring points in the sample set. Next, we calculated a simple derivative of the signal by determining the difference between each pair of consequent points. The pulses were identified from the derivative of the trace using a threshold detection algorithm. The threshold was estimated as the detection accuracy coefficient multiplied by the standard deviation of the derivative of the trace. Suprathreshold points on the derivative of the trace were taken as the beginnings and ends of the pulses (Zakharov et al., 2013).

### Electron Microscopy

Primary hippocampal cultures were fixed in 2.5% glutaraldehyde (Acros Organics, AC119980010, United States) on DIV 10 and DIV 14. The cultures were then washed three times with PBS and treated with 1% osmium tetroxide (Sigma-Aldrich, 20816-12-0) for 60 min. After additional washing steps, the dissociated hippocampal cells were dehydrated in a series of ethanol solutions of increasing concentration (30–100%) followed by 100% acetone and then embedded in a mixture of acetone/EPON resin (50:50). The culture was ultimately embedded in EPON resin (Fluka, United States).

For electron microscopy, the resin blocks were cut using a Leica EM UC7 ULTRA ultramicrotome (Leica, Germany). Ultrathin sections were contrasted with 4% uranyl acetate (SPI-chem, 02624-AB, United States), lead citrate and trihydrate (SPI-chem, 512-26-5). The ultrathin sections were examined with a Morgagni 268D transmission electron microscope (FEI Company, United States).

### Registration of Mitochondrial Functional Activity

Mitochondrial functional activity was analyzed on DIV 10 and DIV 14. Mitochondria were isolated using the standard differential centrifugation method (Pallotti and Lenaz, 2001; Schmitt et al., 2015), and the dissociated hippocampal cells were enzymatically [versine-trypsin (3:1) solution] removed from the cultivation substrate. The subsequent manipulations were performed on ice, and the equipment and isolation media were also cooled. The cells were placed into a precooled porcelain mortar; washed with an ice-cold isolation medium comprising 70 mM saccharose, 210 mM mannitol, 30 mM HEPES, and 0.1 mM EDTA (pH 7.4); and then subjected to homogenization in a glass homogenizer. An electrically driven Teflon pestle had a clearance excluding mitochondrial destruction. The obtained homogenate was centrifuged at 4,000 rpm (temperature ranging from -3 to 0°C) for 10 min. The precipitate was resuspended in medium containing 210 mM mannitol, 70 mM saccharose, 0.1 mM EGTA, and 10 mM HEPES (pH 7.4). The obtained mitochondrial suspension was stored on ice to avoid freezing. The Bradford method was used to quantitate the protein in the isolated mitochondria.

Oxygen consumption by the isolated mitochondria was registered polarographically using the high-resolution respirometer OROBOROS Oxygraph-2k (OROBOROS Instruments, Austria) in 2 mL incubation medium (210 mM mannitol, 70 mM saccharose, 0.1 mM EGTA, 10 mM HEPES, pH 7.4) with constant stirring. The oxygen consumption rate was expressed in picomol/s/1 mg mitochondrial protein.

The oxygen consumption in the chamber was fixed using DatLab5 software (OROBOROS Instruments, Austria).

The state of the mitochondrial respiratory chain was evaluated according to the following parameters: the rate of oxygen consumption by mitochondria with a high substrate content, 5 mM glutamate and 5 mM malate (substrates of complex I), in the incubation medium; the oxidative phosphorylation rate of the respiratory chain in the presence of 5 mM adenosine diphosphate (ADP); the inhibition of complex I activity with 0.5 μM rotenone, and the work intensity of the respiratory chain after the stimulation of complex II with 10 mM sodium succinate.

### Statistical Analysis

All quantified data are presented as the mean ± standard error of the mean (SEM). Statistical analyses were performed using two-way ANOVA implemented in Sigma Plot 11.0 software (Systat Software, Inc.). The Student–Newman–Keuls (SNK) test was used as a *post hoc* test following ANOVA. Differences between groups were considered significant if the corresponding *p*-value was less than 0.05.

## Results

First, we selected an appropriate concentration for the selective TrkB receptor blocker ANA-12. ANA-12 is low-molecular-weight heterocyclic compound that affects the formation of a functional complex between BDNF and TrkB (Cazorla et al., 2011). ANA-12 binds the extracellular fifth subdomain of TrkB (TrkB-d5). Given the size of BDNF relative to ANA-12, although the N-terminal arm of the neurotrophin competes with ANA-12 for the TrkB-d5-binding pocket, it can be assumed that the small compound can easily bind to the active center of TrkB.

A single application of ANA-12 at a concentration of 24 μM exerted a pronounced toxic effect on primary hippocampal cultures. The number of viable cells treated with 24 μM ANA-12 was significantly decreased by 1.3-fold compared to that in the “sham” group (day 7 after the addition; sham: 91.32 ± 2.17; ANA-12: 70.24 ± 3.42). Moreover, even though single ANA-12 applications at concentrations of 12 μM, 10 μM, and 5 μM did not affect cell viability, this daily application caused high primary hippocampal cell mortality by DIV 7. Therefore, the concentration of ANA-12 was reduced to 1 μM. According to previous studies, the use of ANA-12 at this concentration completely negates the neuroprotective effect of BDNF (Cazorla et al., 2011; Longo and Massa, 2013; Saba et al., 2018). These findings indicate that such concentrations of ANA-12 are sufficient to block the majority of TrkB receptors. Application of BDNF, ANA-12 and BDNF and ANA-12 in combination was carried out according to the scheme presented in the “Materials and Methods” section.

To identify the role of TrkB receptors in the formation of neural network activity and the adaptive potential of brain cells in the context of chronic test substance usage, we performed analyses of spontaneous bioelectrical and calcium activities, morphological studies, and evaluations of the dynamics of mitochondrial respiratory chain functional characteristics in neural cells on DIV 7, 10, and 14.

### Features of the Spontaneous Bioelectrical Activity of Neural Networks in Primary Hippocampal Cultures in the Context of Chronic TrkB Receptor System Influence

Electrophysiological data analysis revealed that daily application of the tested substances to the culture medium modulated spontaneous bioelectrical activity in primary hippocampal cultures.

**FIGURE 2:**
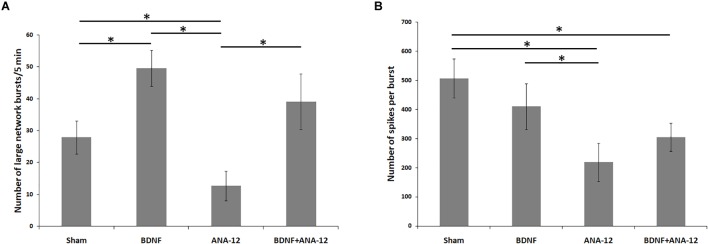
Main parameters of spontaneous bioelectrical activity in primary hippocampal cell cultures on DIV 14. **(A)** Number of large network bursts/5 min; **(B)** number of spikes per burst. ^∗^*p* < 0.05, ANOVA, *N* = 9.

According to the classical concept, a network burst is considered an event comprising no fewer than four spikes simultaneously recorded from different electrodes in a 50 ms interval (Wagenaar et al., 2006; Pimashkin et al., 2011; Vedunova et al., 2013). As the main purpose of this study was to investigate the neural network complex structure, we focused on the events that simultaneously captured the prevailing portion of the functionally active cells. In this regard, all neural network bursts were conditionally divided into small (from 4 to 100 spikes in 50 ms) and large (101 or more spikes in 50 ms) groups (Figure 2 and Supplementary Table 1). The detection of large network bursts allows the identification of the network structure by the cross-correlation method and graphs and the presentation of the neural network activation pattern.

The development of neural network activity is associated with the gradual formation of new contacts between neurons and synapse maturation.

The first network bursts were registered in the “sham” group on DIV 7. Although small network bursts prevailed (approximately 82.3 ± 10.76% of all network bursts), large neural network events were also observed. However, the number of spikes in a burst remained relatively small (number of large bursts/5 min: 34.49 ± 7.32; number of spikes in a large burst: 123.82 ± 18.97). On DIV 10, the number of large network bursts was decreased (number of large bursts/5 min: 21.04 ± 3.21), whereas the number of spikes in a large burst was significantly increased (number of spikes in a large burst: 226.78 ± 41.51). On DIV 14 on the maturing synaptic contact background, an insignificant increase in the number of large bursts and stabilization of bioelectrical parameters were observed (number of large bursts/5 min: 27.87 ± 5.21; number of spikes in a large burst: 506.54 ± 67.11).

Chronically blocking TrkB receptors leads to the modulation of neural network activity. On DIV 14, the number of large bursts was significantly decreased to 12.65 ± 4.65 after TrkB receptors were chronically blocked, and the number of spikes in a large burst was also decreased compared to that in the sham group and amounted to 218.54 ± 65.12.

Notably, the largest number of large network bursts was registered in cultures to which BDNF was applied chronically (number of large bursts/5 min on DIV 14: 49.56 ± 5.67), and the number of spikes in a burst was comparable to that in the sham group (number of spikes in a large burst on DIV 14: 409.65 ± 78.32).

**FIGURE 3:**
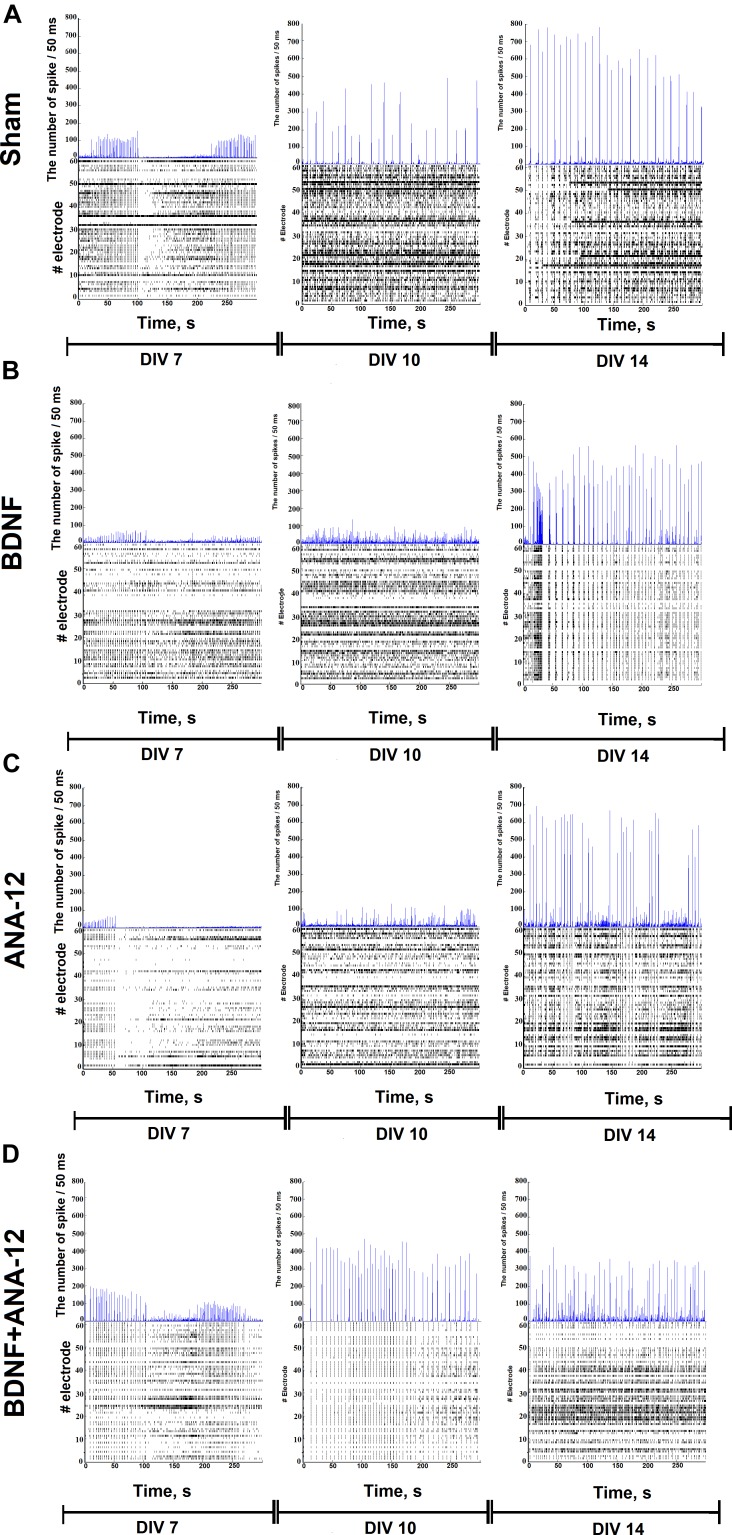
Number of spikes/50 ms and raster diagrams of spontaneous bioelectrical activity in primary hippocampal cultures during development *in vitro*: **(A)** sham, **(B)** BDNF, **(C)** ANA-12, **(D)** BDNF + ANA-12.

The combined application of BDNF and ANA-12 activated neural network activity, which is specifically related to the period of bioelectrical process reformatting and replacement of the main paths of information transmission from electrical to chemical synapses (DIV 10). In this period, the number of large bursts/5 min was 42.34 ± 8.45, and the number of spikes in a large burst was 320.78 ± 59.89. By DIV 14, despite the high number of large bursts (39.08 ± 8.76), no significant increase in the number of spikes in a large burst was observed; this parameter remained the same as that observed on DIV 10 (Figure 3 and Supplementary Figure 1).

The characteristic neural network activation profile showed an increase in the activation time during culture development, which can be attributed to signal transmission through chemical synapses occurring on a time delay and creating complicated neural network structures during developmental processes. The use of BDNF exerts a more pronounced effect on increasing the transmission time of the first signal, which is probably associated with a change in the proportion of different synapses under chronic neurotrophin application. In another experimental group, changes in the pattern activation profile were not observed (see Supplementary Figure 2).

**FIGURE 4:**
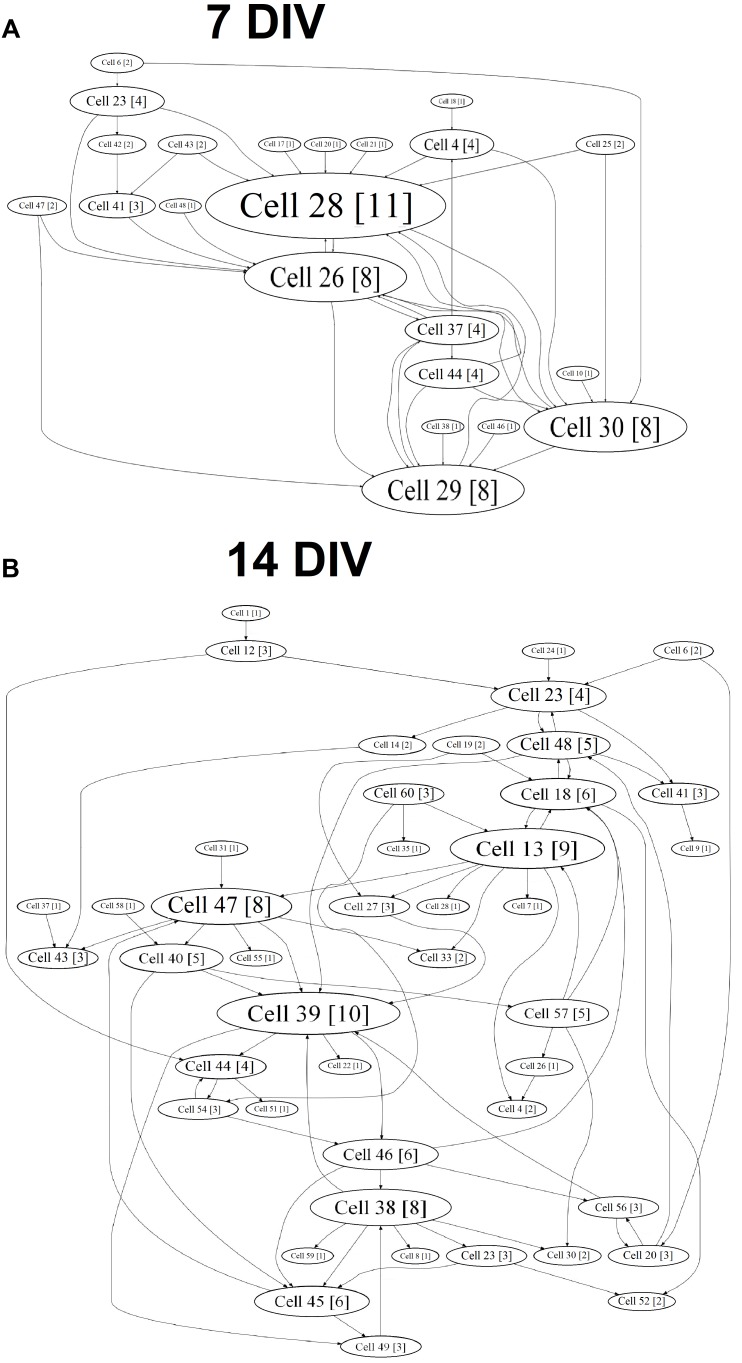
Internal functional structure of neural networks in the primary hippocampal cultures during development *in vitro* (“sham” group): graphical representation of the correlated connections among neurons in the network. The electrode number is presented as “Cell X.” The number of connections on the electrode is indicated in square brackets. The vertex size is proportional to the number of significant connections. **(A)** DIV 7, **(B)** DIV 14.

A cross-correlation analysis showed the complicated neural network structure and the appearance of hubs during the *in vitro* culture development (Figure 4). The gradual formation of a sustainable neural network without the redistribution of activity centers was observed in the “sham” group, and 77.56 ± 17.23% of the connections remained stable from DIV 10 to DIV 14 (Table 1).

**Table 1:** Functional rearrangement alterations in neural networks during primary hippocampal culture development *in vitro.*

Group	Percentages of overlap between DIV 10 and DIV 14
Sham	77.56 ± 17.23%
BDNF	57.37 ± 19.56%
ANA-12	5.75 ± 2.82%
BDNF + ANA-12	26.46 ± 9.8%


Chronically influencing the TrkB receptor system leads to changes in the parameters of spontaneous bioelectrical activity and significantly affects neural network stability. During neural network formation, an active redistribution of the network structure was observed in all experimental groups (percentages of overlap between DIV 10 and DIV 14: BDNF: 57.37 ± 19.56%; ANA-12: 5.75 ± 2.82%; BDNF + ANA-12: 26.46 ± 9.8%) (Table 1).

In addition, an increased number of hubs were observed during the development of primary cultures *in vitro*. This change was apparently related to the complexity of the network structure. The gradual formation of the hub structure and increased significance of individual electrodes are shown for the “sham” and “BDNF” groups. Blocking TrkB receptors decreased the number of hubs in all observation periods (Table 2).

**Table 2:** Number of hubs in the primary hippocampal neural network on different days of development *in vitro.*

Group	DIV 7	DIV 10	DIV 14
Sham	2.08 ± 0.2878	3.4 ± 0.4	6.2778 ± 0.5227
BDNF	2.5 ± 0.4534	3.7273 ± 0.6338	6.0769 ± 0.3294
ANA-12	0.87 ± 0.34*	1.2 ± 0.43*	2.3 ± 0.88*
BDNF + ANA-12	0.76 ± 0.12*	0.67 ± 0.33*	0.45 ± 0.12*


*^∗^ versus “Sham” *p* < 0.05, ANOVA, *N* = 9.*

The same tendency was observed for the parameter involving the number of connections in a hub. During a period of active neural structure formation, increases in the number of connections in a hub were observed in the “sham” and “BDNF” groups (Table 3).

**Table 3:** Average number of connections in a hub in the primary hippocampal neural network on different days of development *in vitro.*

Group	DIV 7	DIV 10	DIV 14
Sham	10.66 ± 0.97	11.13 ± 0.96	12.6667 ± 0.9718
BDNF	10.6667 ± 0.6667	11.03 ± 1.11	13.6250 ± 0.6529
ANA-12	10.11 ± 0.12*	10.23 ± 0.12*	10.57 ± 0.29*
BDNF + ANA-12	10.09 ± 0.23*	10.52 ± 0.57*	10.34 ± 0.54*


*^∗^ versus “Sham” *p* < 0.05, ANOVA, *N* = 9.*

### Features of Spontaneous Calcium Activity in Neural Networks of Primary Hippocampal Cultures in the Context of Chronic TrkB Receptor System Influence

Next, we investigated the features of functional calcium activity in primary hippocampal cell cultures under chronic TrkB receptor system influence.

Registration of calcium dynamics in neural cells using the Ca^2+^ imaging technique is considered the most informative method for studying metabolic neural network activity (Braet et al., 2004; Jercog et al., 2016; Carrillo-Reid et al., 2017). Ca^2+^ is a key regulator of various metabolic processes, and registration of its concentration dynamics in the cytoplasm allows for fine analyses of both neuronal and glial activity.

On DIV 7, spontaneous calcium activity was observed in the “sham” culture, and the percentage of cells that exhibited Ca^2+^ activity was 52.65 ± 3.69% (Supplementary Table 2A). In all experimental groups, this parameter was comparable to the intact values (percentages of cells that exhibited Ca^2+^ activity on DIV 7: BDNF: 51.14 ± 3.05%, ANA-12: 47.98 ± 2.95%, BDNF + ANA-12: 42.97 ± 5.23%).

Beginning on DIV 10, the percentage of cells that exhibited Ca^2+^ activity was significantly lower in the “ANA-12” group than in the “sham” group (percentages of cells that exhibited Ca^2+^ activity on DIV 10: sham: 57.30 ± 3.88%; ANA-12: 35.37 ± 2.39%). The number of active cells in the “BDNF + ANA-12” group was comparable to that in the “BDNF” group (percentage of cells that exhibited Ca^2+^ activity on DIV 10: BDNF: 65.92 ± 3.94%; BDNF + ANA-12: 71.50 ± 3.70%).

**FIGURE 5:**
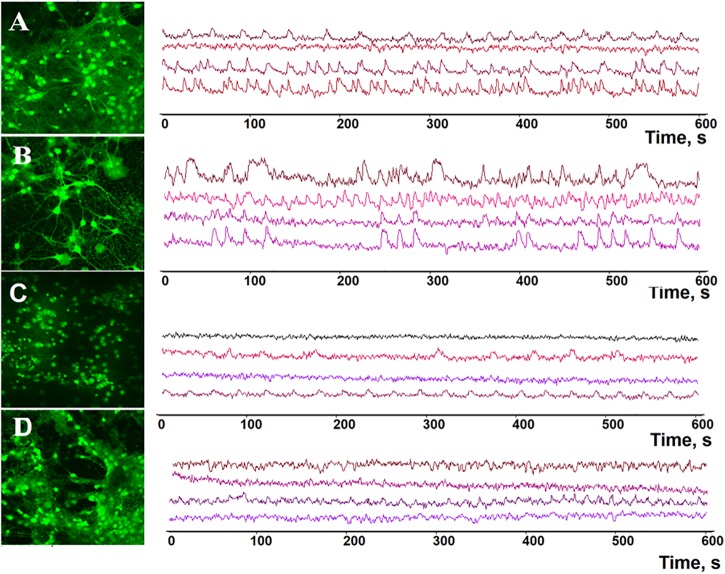
Representative profiles of spontaneous calcium network activity in primary hippocampal cultures on DIV 14. **(A)** Sham, **(B)** BDNF, **(C)** ANA-12, **(D)** BDNF + ANA-12.

**FIGURE 6:**
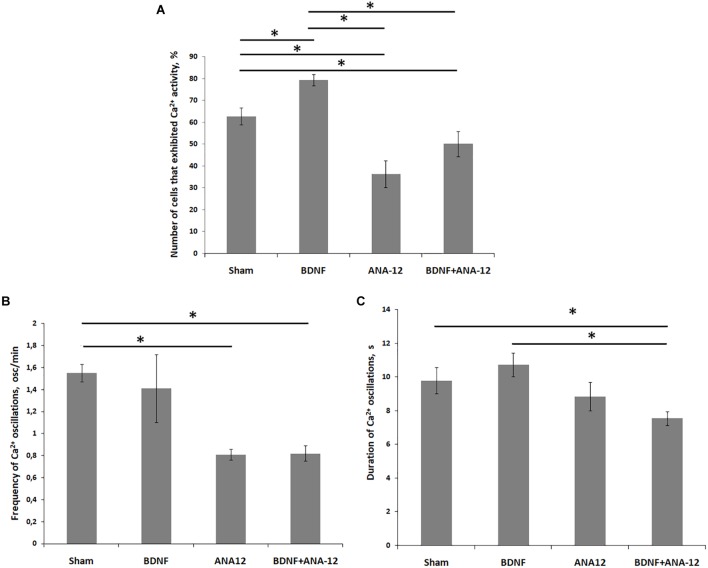
Main parameters of spontaneous calcium activity in primary hippocampal cell cultures during development *in vitro* on DIV 14. **(A)** Proportion of cells exhibiting calcium activity; **(B)** number of Ca^2+^ oscillations per min; **(C)** duration of Ca^2+^ oscillations. ^∗^*p* < 0.05, ANOVA, *N* = 9.

On DIV 14, the percentage of cells exhibiting Ca^2+^ activity in the “BDNF” group was 79.39 ± 2.52%, which significantly exceeded the values in the “sham” (62.77 ± 3.84%), “ANA-12” (36.38 ± 6.06%) and “BDNF + ANA-12” (50.1 ± 5.67%) groups (Figures 5, 6).

During the early stage of culture development (DIV 7), the frequencies of Ca^2+^ oscillations in the “BDNF” and “ANA-12” groups did not differ from the intact values [frequencies of Ca^2+^ oscillations (ocs/min): sham: 0.74 ± 0.09; BDNF: 0.85 ± 0.10; ANA-12: 0.76 ± 0.06]. In the “BDNF + ANA-12” group, this parameter exceeded the intact values by 1.87-fold and amounted to 1.39 ± 0.12 ocs/min (Supplementary Table 2B). A similar change dynamic was observed on DIV 10; however, this parameter was significantly decreased relative to that in the “sham” group on DIV 14 [frequencies of Ca^2+^ oscillations (ocs/min): sham: 1.55 ± 0.08; BDNF + ANA-12: 0.82 ± 0.07]. At this stage of culture development, a decreased frequency of Ca^2+^ events in the “ANA-12” group was also observed (0.81 ± 0.05 ocs/min). In the “BDNF” group, the frequency of Ca^2+^ oscillations was increased on DIV 10. This parameter was normalized to the intact values by day DIV 14 (1.41 ± 0.31 ocs/min) (Figures 5, 6).

The duration of Ca^2+^ oscillations in all experimental groups did not significantly differ from the intact values on DIV 7 (Supplementary Table 2C). From DIV 10, a decrease in this parameter was observed in the “ANA-12” and “BDNF + ANA-12” groups [durations of Ca^2+^ oscillations (s): sham: 9.67 ± 0.69; ANA-12: 7.21 ± 0.47; BDNF + ANA-12: 6.48 ± 0.30]. Chronic BDNF application did not affect the duration of Ca^2+^ oscillations throughout the entire observation period (BDNF on DIV 7: 10.5 ± 0.28; BDNF on DIV 10: 8.46 ± 0.43; BDNF on DIV 14: 10.73 ± 0.71) (Figures 5, 6).

Thus, activation of the TrkB receptor system during development promotes the stimulation of neural network functional calcium activity, which was manifested in the increased number of cells exhibiting calcium activity and the frequency of network calcium events. Blocking TrkB receptors leads to the irreversible suppression of spontaneous calcium activity in primary hippocampal cultures beginning on DIV 10.

### Ultrastructural Features of Neural Networks in Primary Hippocampal Cultures in the Context of Chronic TrkB Receptor System Influence

**FIGURE 7:**
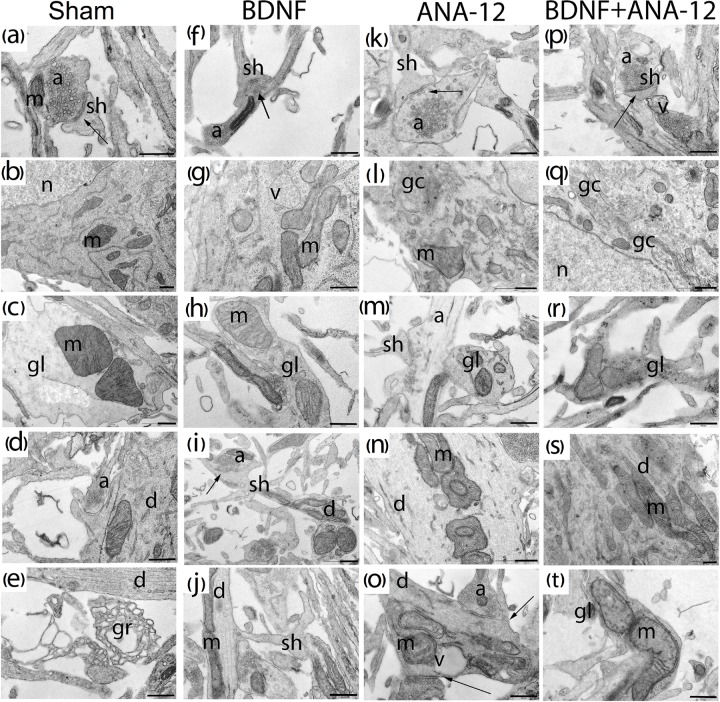
Representative electron microscopy images of dissociated hippocampal cells on DIV 10. **(a–e)** Sham, **(f–j)** BDNF, **(k–o)** ANA-12, **(p–t)** BDNF + ANA-12. **(a)** Axo-spiny synapse; the cristae of mitochondria in a dendrite are smooth. **(b)** Neuronal soma; the cytoplasm shows well-visualized mitochondria, Golgi apparatus, granular endoplasmic reticulum, and numerous free ribosomes. **(c)** Mitochondria in a glial cell; cristae are smooth, and the glia-glial gap junction is well visualized. **(d)** Cisterns of the endoplasmic reticulum in a dendrite are expanded, and the cristae in a mitochondrion are smooth. **(e)** Growth cone. **(f)** Mitochondrion in a neuronal axon. **(g)** Mitochondria in a neuronal soma, some are without cristae and have an enlightened matrix, a vacuole, and numerous free ribosomes. **(h)** Glial outgrowth, a few glycogen pellets and uneven cristae in a mitochondrion are visible. **(i)** Axo-spiny contact; mitochondria in a dendrite have swollen cristae. **(j)** Dendritic spine. **(k)** Axo-spiny synapse, short postsynaptic density (PSD), few synaptic vesicles near the active zone, and large osmiophilic bubbles in an axon. **(l)** Part of the cell body; a destroyed Golgi apparatus is visualized, mitochondria have normal structures, and an extremely low number of ribosomes are present on the endoplasmic reticulum. **(m)** Mitochondria in a glial cell, axonal outgrowth, and an enlightened axoplasm. **(n)** Mitochondria in a dendrite are densely packaged, have an irregular shape and exhibit cristae with small extensions. **(o)** Axo-dendritic synapse, destroyed mitochondria in a dendrite. **(p)** Mature axo-spiny synapse, a vacuole in an axon. **(q)** Increased Golgi apparatus area, a vacuole and destroyed mitochondria in the cytoplasm. **(r)** Mitochondria with impaired internal structures in a glial outgrowth, with a large number of osmiophilic bubbles. **(s)** Mitochondria in a large dendrite. **(t)** Mitochondria in a glial outgrowth have impaired internal structures. Scale bar – 0.5 μm.

**FIGURE 8:**
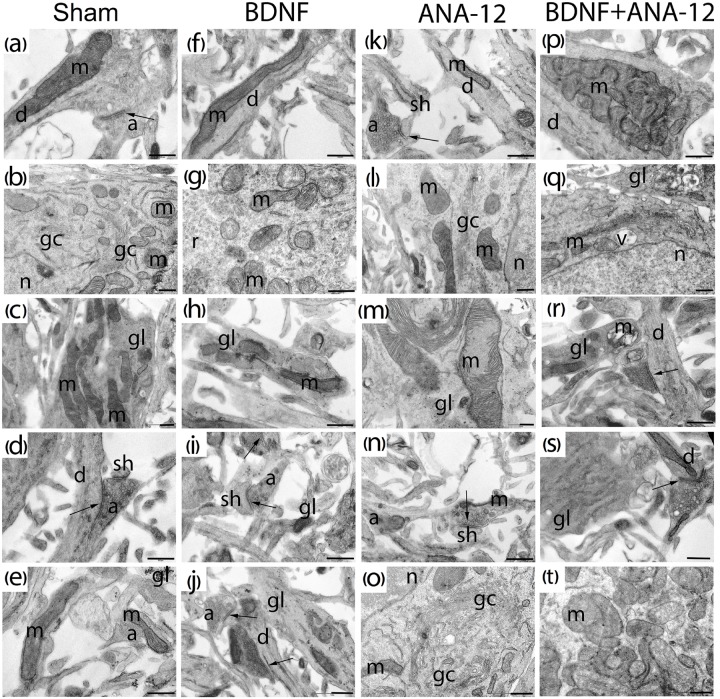
Representative electron microscopy images of dissociated hippocampal cells on DIV 14. **(a–e)** Sham, **(f–j)** BDNF, **(k–o)** ANA-12, **(p–t)** BDNF + ANA-12. **(a)** Axo-spiny synapse; the cristae of mitochondria in a dendrite are smooth. **(b)** Cytoplasm of a neuron; mitochondria, Golgi apparatus, granular endoplasmic reticulum, numerous ribosomes and part of the nucleus are well visualized. **(c)** Mitochondria in a glial cell; cristae are smooth, and intermitochondrial contacts are visible. **(d)** Axo-dendritic and axo-spiny asymmetric synapse. **(e)** Immature axo-spiny synapse; no postsynaptic density is observed. **(f)** Mitochondrion in the neuronal dendrite. **(g)** Mitochondria in a neuronal soma; some are without cristae and have an enlightened matrix and short cristae. **(h)** Glial outgrowth with few glycogen pellets and irregular cristae. **(i)** Axo-spiny synapse; osmiophilic synaptic bubbles in an axon. **(j)** Axo-dendritic and axo-spiny asymmetric synapses, osmiophilic mitochondrion in an axon; mitochondria in the axonal outgrowth are destroyed. **(k)** Axo-spiny asymmetric synapse, short postsynaptic density. **(l)** Part of a neuronal soma, destroyed Golgi apparatus, few ribosomes on the endoplasmic reticulum, and normal mitochondrial structure. **(m)** Mitochondria with disrupted cristae in a glial cell. **(n)** Axo-spiny asymmetric synapse, osmiophilic bubbles among synaptic vesicles, mitochondrion with an irregular shape and high osmiophility. **(o)** Part of a neuronal soma, Golgi apparatus, and mitochondria with impaired structures. **(p)** Mitochondrial cluster in a dendrite. **(q)** Golgi apparatus with an increased area, a vacuole and destroyed mitochondria in the cytoplasm. **(r)** Axo-dendritic synapse and vacuolated mitochondria in a glial outgrowth. **(s)** Glial cell with a smooth endoplasmic reticulum, synaptic vesicles of different sizes in the axon, and mitochondrion with a reduced area. **(t)** Mitochondrial clusters in the neuronal cytoplasm; many of these clusters have extended cristae; mitochondria without cristae were also visualized. Scale bar – 0.5 μm.

Qualitative and quantitative ultrastructural analyses of primary hippocampal cell cultures performed on DIV 10 and DIV 14 allowed the identification of culture organization features in the context of chronic TrkB receptor system influence (Figures 7, 8).

Many axonal buds containing equal-sized synaptic vesicles and dendritic spines without contacts were observed in the “sham” group on DIV 10. At this stage of culture development, a few mature symmetric and asymmetric contacts were observed. Furthermore, glycogen granules were visualized in glial outgrowths, and many cones of growth were observed (Figures 7a–e).

Previous studies have shown that a single BDNF application affects the functional and ultrastructural characteristics of neural and glial brain mitochondria (Markham et al., 2012). The current study did not reveal significant changes in the mitochondrial apparatuses (occupied area or amount or ultrastructural organization) of primary hippocampal cells chronically administered BDNF, ANA-12 or BDNF and ANA-12 in combination. The mitochondrial structure typically exhibits smooth cristae and an osmiophilic matrix, and the organelle shape is oval. However, in the “BDNF” group, insignificant changes in these parameters were found. In addition, numerous glycogen granules in glial outgrowths as well as numerous ribosomes in the granular endoplasmic reticulum and free ribosomes were visualized (Figures 7f–j).

**FIGURE 9:**
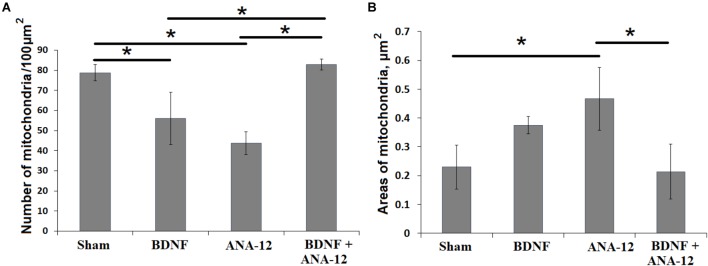
Number of mitochondria **(A)** and their areas in the neuronal soma **(B)** on day 14 of culture development *in vitro*. ^∗^*p* < 0.05, ANOVA, *N* = 3.

Significant changes in the mitochondrial apparatus structure were observed in the “ANA-12” group. Mitochondria with impaired internal organization were visualized in the view field. However, no quantitative changes in the occupied area or the number of mitochondria per μm^2^ of neuronal soma were detected (Figure 9). Mitochondria with modified shapes and intermitochondrial contacts in the outgrowth were also observed. In this group of cultures, axonal cytoskeleton violations were visualized, and “empty” axons containing only synaptic vesicles were identified in the observation field. Delayed synaptogenesis was also observed.

Mature synapses had weak postsynaptic density (PSD) osmiophility, and “contacts” between the axonal bud and a dendrite or dendritic spine in the absence of PSD were often observed. A distinctive feature of the ultrastructural organization under a chronic TrkB receptor blockade was the cistern expansion in the smooth endoplasmic reticuli of glial and neural outgrowths. Reconstruction of the Golgi apparatus lamellar complex and its cistern overgrowth were observed in the cell body (Figures 7k–o).

In contrast to the “sham” group, the “BDNF + ANA-12” group exhibited disrupted mitochondria and mitochondria with impaired or vacuolated cristae. Nevertheless, numerous synaptic contacts and a well-defined PSD were observed in “BDNF + ANA-12” cultures compared to those in “ANA12” cultures (Figures 7p–t).

No significant differences in the number of mature synaptic contacts were observed on DIV 14 in any experimental groups (Figure 8). Thus, delayed synaptogenesis in the “ANA12” group was overcome on DIV 14. This delay was presumably associated with the cell synthetic apparatus because weak PSD was detected on DIV 10, although PSD is normally formed first during cell culture ontogenesis (Meyer et al., 2014).

**FIGURE 10:**
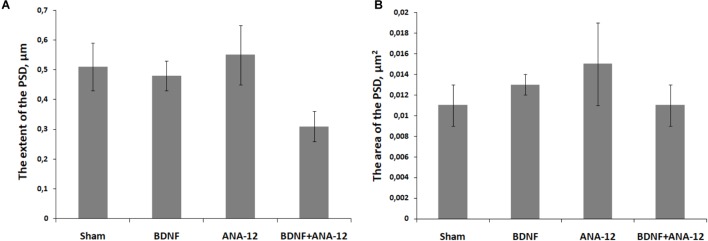
The extent **(A)** and area **(B)** of the PSD in postsynaptic asymmetric contacts on day 14 of culture development *in vitro*.

Analysis of PSD features in postsynaptic asymmetric contacts under chronic administration of the tested substances was performed on DIV 14 (Figure 10). We did not find significant changes in the area or extent of the PSD. However, the extent of the PSD in the “BDNF + ANA-12” group had a tendency to decrease (sham: 0.511 ± 0.31, BDNF + ANA-12: 0.32 ± 0.13). These findings could be explained by the high variability of synaptogenesis at this stage of development. Because the synaptogenic processes had not been completed by DIV 14, we observed synapses with various sizes and characteristics. Synapses in the “BDNF + ANA-12” group were short (Figures 8p–t), and changes in the presynaptic terminal were also observed.

**FIGURE 11:**
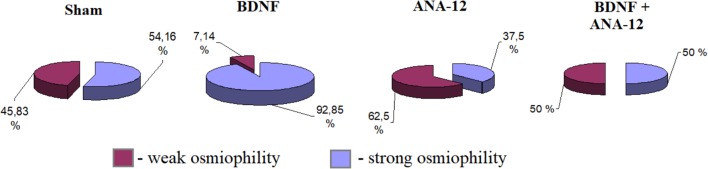
Percentages of the numbers of postsynaptic densities with differing osmiophilities in asymmetric synapses on day 14 of culture development *in vitro*. Red – weak osmiophility, blue – strong osmiophility.

An analysis of PSD osmiophility in asymmetric synapses revealed that the percentage of high osmiophilic PSD in the “BDNF” group was increased by 38.69% compared to that in the “sham” group, which is potentially indicative of more efficient signal transmission between neurons (Figure 11). By contrast, a delay in synaptogenesis was observed in the “ANA-12” group, and 16.16% fewer synaptic contacts were observed compared to intact cultures. Despite the reduced PSD length, the osmiophility in the “BDNF + ANA-12” group was comparable to that in the “sham” group.

The number of mitochondria in the neuronal soma in the “ANA-12” group was significantly decreased compared to that in the “sham” group and amounted to 43.73 ± 5.66 per 100 μm^2^ (Figure 9A). These changes are associated with organelle destruction. In parallel, increased individual mitochondria areas were observed in the “ANA-12” group (Figure 9B).

Thus, TrkB receptor blockade led to significant ultrastructural changes in primary hippocampal cell cultures, which was primarily associated with alterations in the structure of the mitochondrial cell apparatus. The absence of PSD in the synaptic contact structure was also observed during the early stages of culture development. The number of mature synaptic contacts during synaptogenesis was normalized in later stages of cultivation.

### Mitochondrial Functional Activity in Primary Hippocampal Cell Cultures in the Context of Chronic TrkB Receptor System Influence

Changes in the number of mitochondria and their structure upon influencing the TrkB receptor system are of particular interest. In this regard, the final stage of our study was devoted to the assessment of dynamic changes in the functional characteristics of the mitochondrial respiratory chain in primary hippocampal cells in response to chronic BDNF and ANA12 administration to the culture medium.

**FIGURE 12:**
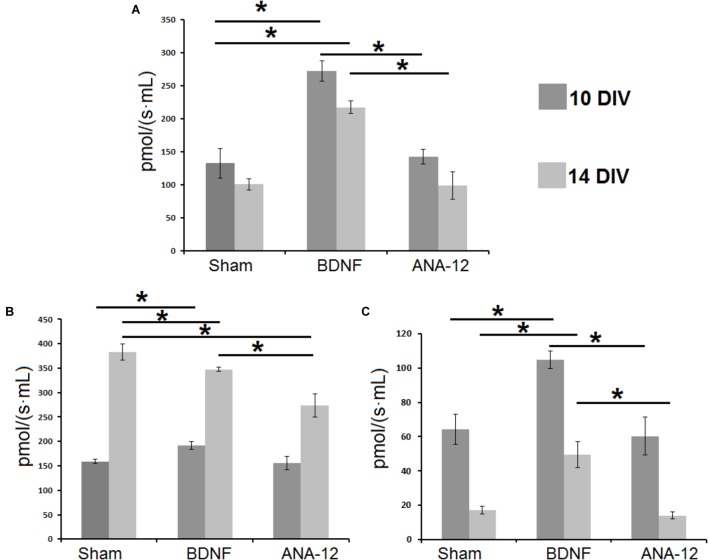
Mitochondrial functional activity in primary hippocampal cell cultures on DIV 10 and DIV 14. **(A)** Basal oxygen consumption rates by mitochondria in primary hippocampal cell cultures. **(B)** Mitochondrial oxygen consumption rates during mitochondrial respiratory chain complex I work. **(C)** Mitochondrial oxygen consumption rates during mitochondrial respiratory chain complex II work. ^∗^*p* < 0.05, ANOVA, *N* = 3.

Daily BDNF application increased the mitochondrial oxygen consumption rate (Figure 12A). The basal oxygen consumption rate in the “BDNF” group significantly exceeded that in the “sham” and “ANA-12” groups and amounted to 272.2 ± 11.5 pmol/(s^∗^mL) on DIV 10 and 217.1 ± 20.5 pmol/(s^∗^mL) on DIV 14. The basal oxygen consumption rate in the “ANA-12” group did not differ from that in the sham group throughout the entire observation period.

In addition, neural-glial network development is associated with increased mitochondrial respiratory chain complex I activity (Figure 12B). On DIV 14, the mitochondrial respiratory chain activity in the “sham” group was 383 ± 17.1 pmol/(s^∗^mL), which was increased by 2.4-fold compared to that on DIV 10 [159.1 ± 3.9 pmol/(s^∗^mL)]. Similar tendencies were observed in the “BDNF” and “ANA-12” groups. However, on DIV 14, the mitochondrial respiratory chain complex I activities in these experimental groups were significantly lower than the intact values. Thus, blocking TrkB receptors leads to a marked decrease in mitochondrial respiratory chain activity. The oxygen consumption rate in the “ANA-12” group was 273.9 ± 23.8 pmol/(s^∗^mL), which was significantly lower than that in the “BDNF” [347.3 ± 4.0 pmol/(s^∗^mL)] and “sham” [383 ± 17.1 pmol/(s^∗^mL)] groups.

Alternatively, the intensity of mitochondrial respiratory chain complex II activity was decreased during culture development. In contrast, chronic BDNF administration increased the respiratory complex II activity (Figure 12C). The oxygen consumption rates in the “BDNF” group on DIV 10 and DIV 14 were 104.9 ± 5.1 pmol/(s^∗^mL) and 49.5 ± 7.5 pmol/(s^∗^mL), respectively. This parameter in the “ANA-12” group did not differ from the intact values.

Thus, BDNF application stimulates mitochondrial functional activity in primary hippocampal cell cultures and manifested as increased mitochondrial respiratory chain complexes I and II activity and mitochondrial oxygen consumption rates. Blocking TrkB receptors leads to marked decreases in mitochondrial respiratory chain complex I activity during late stages of culture development.

## Discussion

Brain activity is associated with complex signal transmission mechanisms and modulation in the neuron-glial network. This complex activation pattern and inhibitory signals summarized in the transmission of information through the neuron-glial network makes it possible to implement complex cognitive and behavioral responses. Understanding the fundamental laws of brain function is currently the main task in modern science and will contribute to solutions for a number of clinical and scientific issues.

In our study, we modeled chronic activation and inhibition of the TrkB receptor system, which is one of the most important systems for neurons. This approach can potentially be used to uncover the basic mechanisms underlying emergent information functions. The application of MEAs and the complexity of functional and ultrastructural studies allowed us to detect changes in the neural network structure under the influence on the TrkB receptor system and to suggest the main intracellular targets for these implemented changes.

The TrkB receptor system is responsible for a number of functions associated with development and brain cell adaptation to unfavorable factors (Patapoutian and Reichardt, 2001; Skaper, 2018). In this case, identifying whether metabolic changes are possible at the neural network activity level is difficult. MEAs are considered the most adequate method for estimating the neural network structure both *in vivo* and *in vitro* (Martinoia et al., 2005; Obien et al., 2014). The application of this technology in combination with mathematical methods of biological data analysis allowed us to identify the features of signal transmission throughout the network and reveal possible mechanisms underlying the systemic effect of chronic BDNF administration and a TrkB receptor system blockade.

Electrical synapses with a small number of immature chemical axo-dendritic and axo-spiny synapses are predominant in the early stages of primary hippocampal cell culture development (Shirokova et al., 2013). During excitation transmission through electrical synapses, signal modulation is limited and does not strongly depend on chemical stimuli (Hormuzdi et al., 2004). However, the influence on the TrkB receptor system can significantly impact chemical contact maturation and eventually affect neural network activity.

A sufficiently high level of spontaneous bioelectrical activity in primary hippocampal cultures in which chronic TrkB receptor blockade is implemented on DIV 14 is possibly associated with long-term adaptation effects related to changes in TrkB receptor expression under chronic influence (Proenca et al., 2016). ANA-12 is low-molecular-weight heterocyclic compound that affects the formation of a functional complex between BDNF and TrkB (Cazorla et al., 2011). The result of ANA-12 binding to the extracellular domain of TrkB is the prevention of BDNF-induced TrkB activation and negation of the biological effects of BDNF on TrkB-expressing cells. The N-terminal region of BDNF, which is critical for selectively binding and activating cognate Trk receptors, interacts with the “specificity patch” of the receptor, a binding pocket located in the fifth subdomain of TrkB (TrkB-d5) that may drive the selectivity of the interaction with BDNF. ANA-12 specifically binds to TrkB-d5 in a dose-dependent manner. Based on previous binding experiments, even high concentrations of ANA-12 cannot overcome its displacement from TrkB by BDNF, suggesting a non-competitive mechanism (Banfield et al., 2001; Pattarawarapan and Burgess, 2003; Cazorla et al., 2011). Thus, the stereometric features of ANA-12 allow the rapid and reliable binding of this molecule to the active center of TrkB, which prevents the biological effect of BDNF.

However, the cross-correlation method and graphs revealed the nearly complete absence of hubs in the network structure under chronic blockade of TrkB receptors. This result is especially noteworthy in the context of high absolute values of spontaneous bioelectrical activity.

Analysis of functional calcium activity showed that changes in the percentages of working cells in the different groups were proportional to changes in the number of spikes in a large burst. The highest percentage of cells exhibiting Ca^2+^ activity was found in cultures chronically administered BDNF. In this experimental group, the highest number of spikes in a large burst was detected on DIV 14. In addition, functional Ca^2+^ imaging confirmed that the marked effects on the TrkB receptor system were manifested in the developmental period associated with chemical synapse predominance.

Ultrastructural changes demonstrated that blocking TrkB receptors affected the structures of synapses (empty axons in neurons, absence of PSD in a synapse) and the mitochondrial apparatus. For a long period, mitochondria were not considered capable of participating in synaptic signal modulation. Mitochondria are considered responsible for only energy metabolism in cells, and their effects are limited by the amount of adenosine triphosphate (ATP) produced. Whether BDNF influences mitochondria remains unclear. According to the present study, TrkB-mediated signaling can affect both the ultrastructural and functional parameters of brain mitochondria, even in normal oxygen and nutrient supply conditions. Chronic TrkB receptor blockade leads to destructive ultrastructural changes in mitochondria wherein the functional activity of organelles is not significantly decreased. Long-term application of BDNF increases the enzymatic activity of the mitochondrial apparatus, but this alteration is not associated with changes in the ultrastructure of organelles. Our findings likely indicate that TrkB-mediated mitochondrial regulation is related to functional modifications of the enzymatic systems in the respiratory chain but not to structural rearrangements of organelles. In this regard, the fundamental question for further research is whether the TrkB-mediated pathway for influencing mitochondria is generalized (that is, carried out through nuclear genes) or directed toward an isolated organelle.

Notably, chronic BDNF application increased the basal oxygen consumption rate via activating respiratory chain complex II. However, according to classical ideas, this pathway is typical utilized in hypoxic states (Kristián, 2004). Under oxygen stress conditions, BDNF increases the adaptive potential by impacting the functional parameters of the mitochondrial apparatus. In the current study, the percentage of osmiophilic PSDs in asymmetrical synapses in the “BDNF” group was significantly higher than that in the “sham” group, which potentially indicates more efficient signal transmission between neurons.

A genetically determined high level of BDNF can provide significant adaptive potential to the nervous system, and its neuroprotective effects are induced via the TrkB receptor system (Yu et al., 2013; Katsu-Jiménez et al., 2016). The current study showed that BDNF-mediated TrkB system activation is responsible for the formation of more complex functionally active neural networks with a high level of synaptic transmission efficiency. Thus, the TrkB signaling system can play a key role in inducing higher cognitive functions.

## Author Contributions

TM, EM, and MV conceived and designed the experiments, analyzed the data, and wrote the manuscript. AU, NV, TA, IK, and OS carried out the experiments. MV supervised the project, conceptualized the original idea, and ensured the financing of the project.

## Conflict of Interest Statement

The authors declare that the research was conducted in the absence of any commercial or financial relationships that could be construed as a potential conflict of interest.
