# Supplementary material for: Features of Neural Network Formation and Their Functions in Primary Hippocampal Cultures in the Context of Chronic TrkB Receptor System Influence
Source: Front Physiol. 2019 Jan 10;9:1925. doi: 10.3389/fphys.2018.01925 (PMC6335358; doi:10.3389/fphys.2018.01925)
Supplement: Supplementary file 1 [file Table_1.DOCX]

**Table 1. Main parameters of spontaneous bioelectrical activity in primary hippocampal cell cultures**

| **Parameter** | **Group** | **DIV 7** | **DIV 10** | **DIV 14** |
| --- | --- | --- | --- | --- |
| Number of large network bursts/5 min | Sham | 34.49±7.32 | 21.04±3.21 | 27.87±5.21 |
|  | BDNF | 5.1±3.12* | 10.12±4.65* | 49.56±5.67* |
|  | ANA-12 | 2.1±1.52* | 13.45±3.96* | 12.65±4.65* |
|  | BDNF+ANA-12 | 39.64±6.15 | 42.34±8.45* | 39.08±8.76 |
| Number of spikes per burst | Sham | 123.82±18.97 | 226.78±41.51 | 506.54±67.11 |
|  | BDNF | 101.51±25.98 | 105.62±21.54* | 409.65±78.32 |
|  | ANA-12 | 102.13±23.59 | 110.76±18.87* | 218.54±65.12* |
|  | BDNF+ANA-12 | 115.76±17.67 | 320.78±59.89 | 304.75±49.14* |

* versus “Sham”, p < 0.05, ANOVA, N=9

**Table 2. Main parameters of spontaneous calcium activity in primary hippocampal cell cultures during development *in vitro***

***A. Number of cells that exhibited Ca^2+^ activity, %***

|  | DIV 7 | DIV 10 | DIV 14 |
| --- | --- | --- | --- |
| Sham | 52.65±3.69 | 57.30±3.88 | 62.77±3.84 |
| BDNF | 51.14±3.05 | 65.92±3.94 | 79.39±2.52* |
| ANA-12 | 47.98±2.95 | 35.37±2.39*# | 36.38±6.06*# |
| BDNF+ANA-12 | 42.97±5.23 | 71.50±3.70* | 50.1±5.67*# |

* versus “Sham”; # versus “BDNF”, p < 0.05, ANOVA, N=9

***B. Frequency of Са^2+^ oscillations, osc/min***

|  | DIV 7 | DIV 10 | DIV 14 |
| --- | --- | --- | --- |
| Sham | 0.74±0.09 | 0.92±0.06 | 1.55±0.08 |
| BDNF | 0.85±0.10 | 1.31±0.08* | 1.41±0.31 |
| ANA12 | 0.76±0.06 | 0.74±0.05 | 0.81±0.05* |
| BDNF+ANA-12 | 1.39±0.12*# | 1.76±0.34* | 0.82±0.07* |

* versus “Sham”; # versus “BDNF”, p < 0.05, ANOVA, N=9

***C. Duration of Са^2+^ oscillations, s***

|  | DIV 7 | DIV 10 | DIV 14 |
| --- | --- | --- | --- |
| Sham | 9.68±0.57 | 9.67±0.69 | 9.80±0.78 |
| BDNF | 10.5±0.28 | 8.46±0.43 | 10.73±0.71 |
| ANA12 | 10.37±0.17 | 7.21±0.47* | 8.84±0.84 |
| BDNF+ANA-12 | 11.58±0.60 | 6.48±0.30*# | 7.55±0.41*# |

* versus “Sham”; # versus “BDNF”, p < 0.05, ANOVA, N=9

**Figure 1.** Representative examples of spontaneous bioelectrical activity recordings in primary hippocampal cultures on day 14 of culture development *in vitro*: (А) sham, (B) BDNF, (C) ANA-12, (D) BDNF+ANA-12.

**Figure 2.** Representative examples of the activation patterns of spontaneous bioelectrical activity in primary hippocampal cultures during development *in vitro*: (А) sham, (B) BDNF, (C) ANA-12, (D) BDNF+ANA-12. The color scale corresponds to the occurrence of the first spike in the network burst and is presented in squares according to the electrodes in the multielectrode array.
