# Supplementary figures and images for: Features of Neural Network Formation and Their Functions in Primary Hippocampal Cultures in the Context of Chronic TrkB Receptor System Influence
Source: Front Physiol. 2019 Jan 10;9:1925. doi: 10.3389/fphys.2018.01925 (PMC6335358; doi:10.3389/fphys.2018.01925)

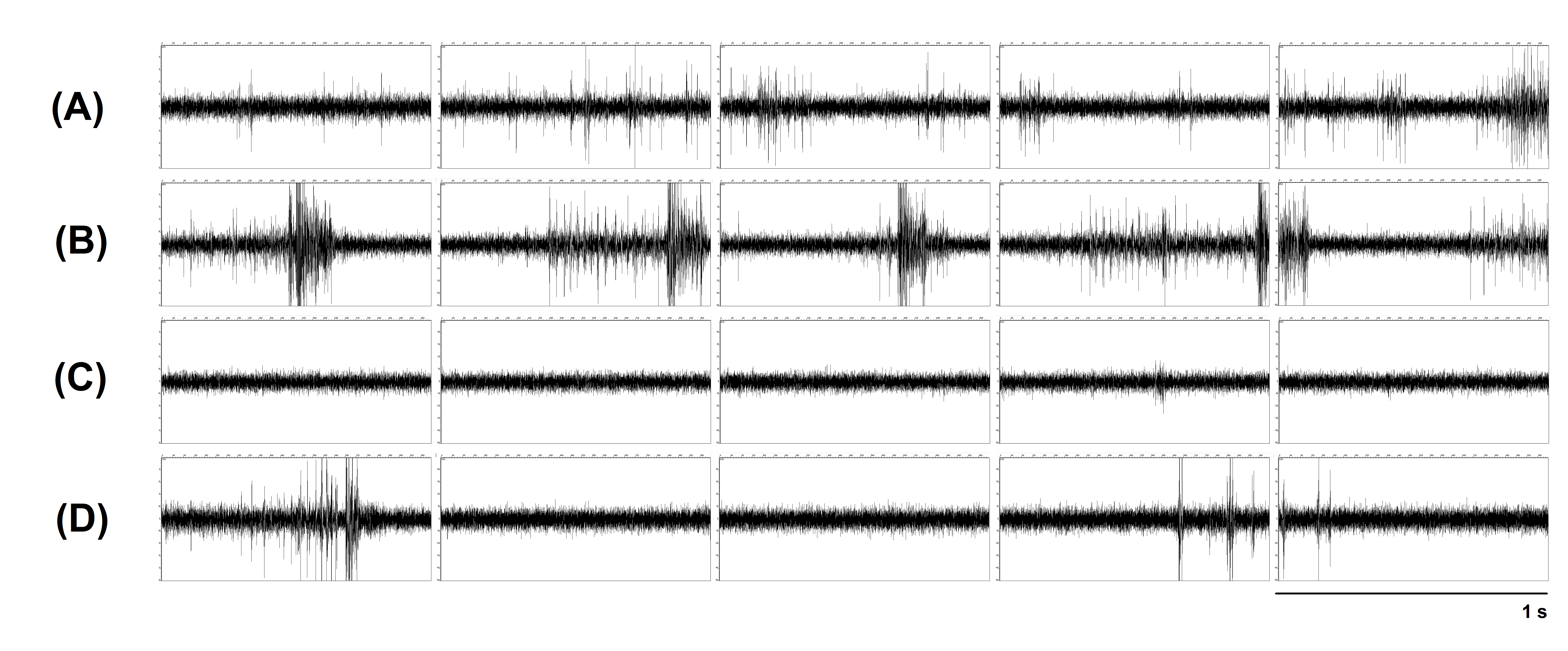

Supplement: Supplementary file 2 [file Image_1.TIF]

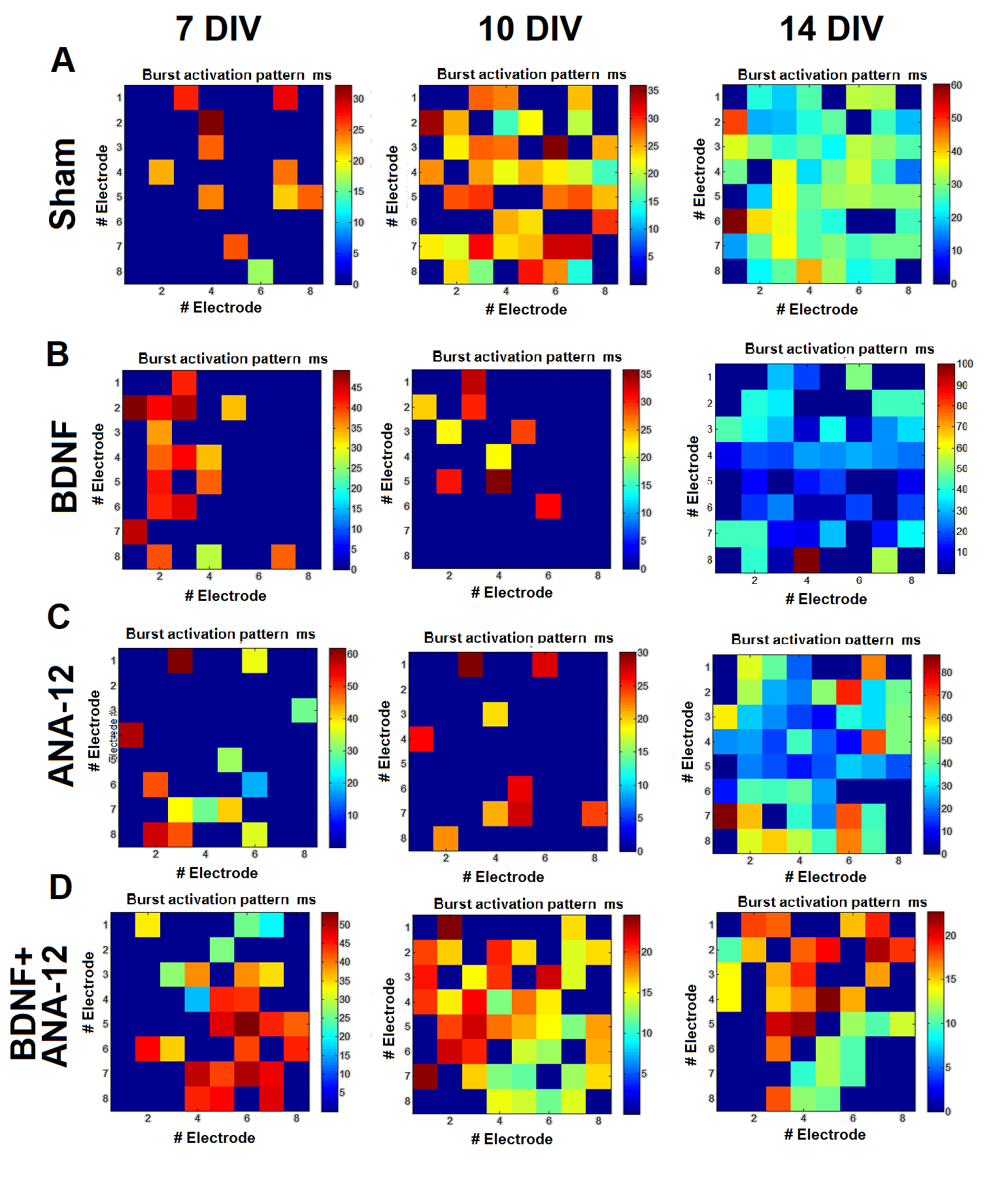

Supplement: Supplementary file 3 [file Image_2.TIF]
